# An emerging generation of endocrine therapies in breast cancer: a clinical perspective

**DOI:** 10.1038/s41523-023-00523-4

**Published:** 2023-04-05

**Authors:** Rima Patel, Paula Klein, Amy Tiersten, Joseph A. Sparano

**Affiliations:** Department of Medicine, Division of Hematology and Medical Oncology, Icahn School of Medicine at Mount Sinai, Tisch Cancer Institute, New York, NY USA

**Keywords:** Drug development, Breast cancer, Targeted therapies

## Abstract

Anti-estrogen therapy is a key component of the treatment of both early and advanced-stage hormone receptor (HR)-positive breast cancer. This review discusses the recent emergence of several anti-estrogen therapies, some of which were designed to overcome common mechanisms of endocrine resistance. The new generation of drugs includes selective estrogen receptor modulators (SERMs), orally administered selective estrogen receptor degraders (SERDs), as well as more unique agents such as complete estrogen receptor antagonists (CERANs), proteolysis targeting chimeric (PROTACs), and selective estrogen receptor covalent antagonists (SERCAs). These drugs are at various stages of development and are being evaluated in both early and metastatic settings. We discuss the efficacy, toxicity profile, and completed and ongoing clinical trials for each drug and highlight key differences in their activity and study population that have ultimately influenced their advancement.

## Introduction

Anti-estrogen therapy targeting the estrogen-mediated signaling pathway is an essential component of treatment for both early and advanced-stage breast cancer expressing the estrogen receptor (ER) and/or progesterone receptor (PR)^1,2^. The ER is a steroid hormone nuclear receptor consisting of a DNA-binding domain (DBD), ligand-binding domain (LBD), and transcriptional activation function domains 1 (AF1) and 2 (AF2). Activated ER can interact with estrogen-responsive elements (EREs) within the DNA through its DBD or interactions with other transcription factors^3^. ER expression occurs in the normal ductal epithelium and invasive breast cancer, and immunohistochemistry can be used to semi-quantitatively measure the degree of ER and PR expression in tumor tissue^4^. Approximately 70% of all breast cancers exhibit ER and/or PR expression and, therefore, potentially sensitive to agents targeting the estrogen signaling pathway, also commonly referred to as “endocrine therapy” (ET)^5^.

For the past 30 years, ET for the treatment of ER-positive metastatic breast cancer (MBC) has generally included selective estrogen receptor modulators (SERMs, e.g., oral tamoxifen), aromatase inhibitors (AIs, e.g., oral anastrozole, letrozole, exemestane), and selective estrogen receptor degraders/downregulators (SERDs, e.g., intramuscular fulvestrant). Tamoxifen, AIs, or ovarian function suppression plus AIs are also effective in reducing recurrence risk when used as adjuvant therapy after primary surgical treatment of localized disease. Of note, AIs have demonstrated superior efficacy compared to tamoxifen, likely due to the agonist activity of tamoxifen, which limits its effectiveness^6,7^. Combination of CDK 4/6 inhibitors with ET has been shown to improve objective response rate (ORR), progression-free survival (PFS), and overall survival (OS) in ER-positive MBC, whether added to an aromatase inhibitor (AI) for first-line ET or fulvestrant as second-line ET after progression or relapse on an AI^8–13^. The CDK 4/6 inhibitor abemaciclib has also been shown to reduce recurrence risk when added to adjuvant AI therapy in those with localized disease at high risk of recurrence^14^. ET combined with agents targeting the PI3K-AKT-mTOR pathway, specifically the mTOR inhibitor everolimus and PI3K inhibitor alpelisib, in the metastatic setting has demonstrated improvements in PFS compared with ET alone^15,16^.

Although most ER-positive breast cancers benefit from ET, some exhibit primary intrinsic resistance, defined as disease progression within 6 months of initiating ET for MBC or relapse within 2 years of initiating adjuvant ET for early breast cancer (EBC). Secondary endocrine resistance, defined as progression ≥6 months after initiating ET for MBC, ultimately develops in most patients. Relapse while on adjuvant ET but after the first 2 years or within 1 year of completing adjuvant ET is also commonly characterized as acquired secondary resistance^5,17^. Secondary resistance to AI therapy is often associated with mutations in the ligand-binding domain of Estrogen Receptor 1 (*ESR1*) that confers ligand-independent activation of ERα^18^. *ESR1* mutations occur in up to 50% of patients receiving AI therapy for MBC and in some receiving adjuvant ET and may be detected by blood using assays that identify circulating tumor DNA (ctDNA)^19^. *ESR1* mutations often occur concurrently with other genomic alterations, which collectively are associated with a worse prognosis^20^. As described in the PADA-1 trial, among patients with baseline *ESR1* mutations and on AI and CDK 4/6 inhibitor therapy for MBC, up to 27% can develop a rise in *ESR1* mutation based on ctDNA at a median time of 15.6 months^21^. Other resistance mechanisms that may be implicated in primary or secondary resistance to ET include *ESR1* loss, amplification, and translocation, and activating alterations in the PI3K-AKT-mTOR, RAS-MAPK, and CDK4/6-RB-E2F pathways, some of which may also contribute to resistance to CDK4/6 inhibitors^18^.

**Fig. 1:**
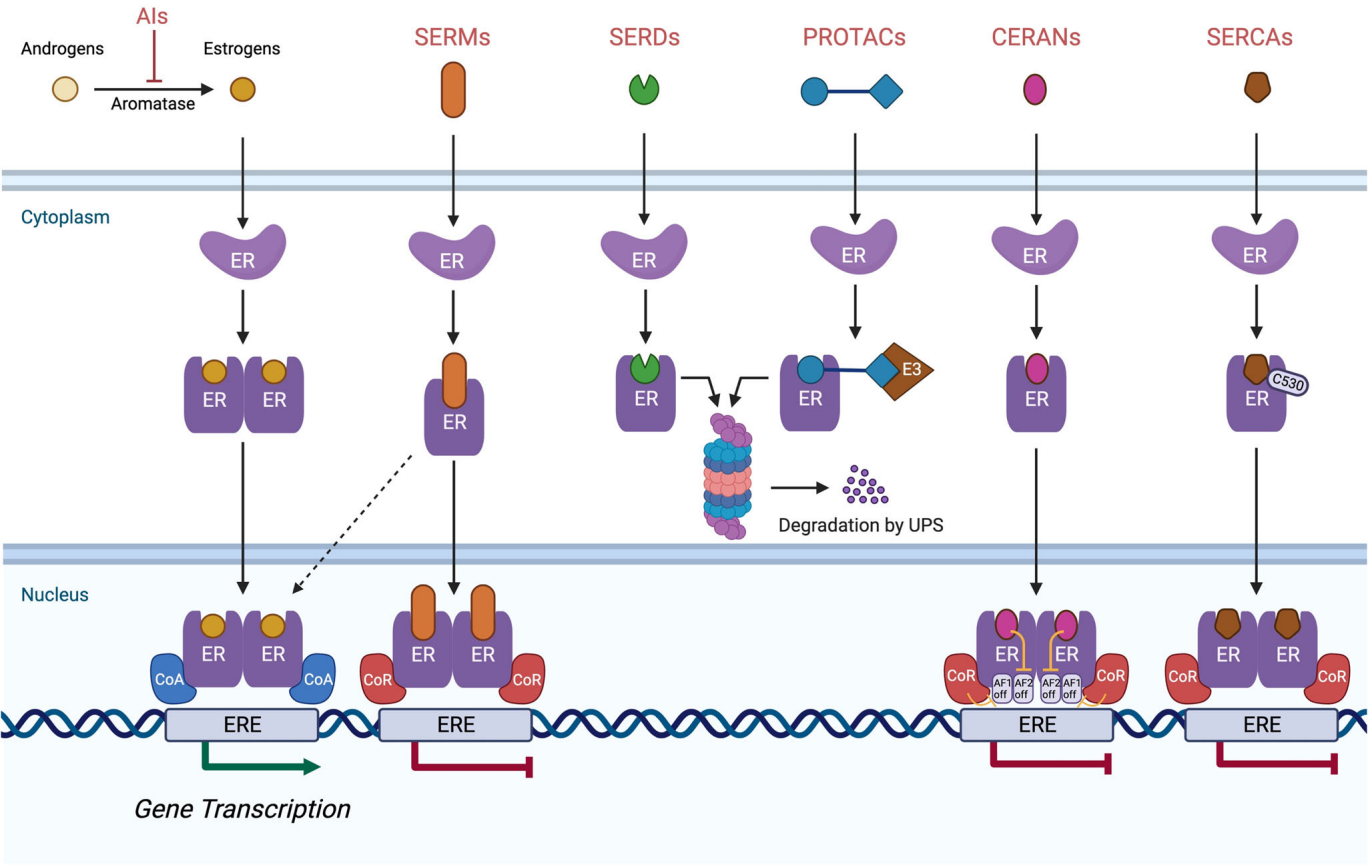
Mechanisms of action of various classes of anti-estrogen therapies. The binding of estrogen to the ligand-binding domain of ER induces an activating conformational change enabling its dimerization and intranuclear localization. Activated ER can interact with estrogen-responsive elements (EREs), allowing for gene transcription, which leads to cell survival and proliferation. *Aromatase inhibitors (AIs)*. AIs, block estrogen production by inhibiting aromatase, which converts androgens to estrogens. *Selective estrogen receptor modulators (SERMs)*. SERMs competitively inhibit the binding of estrogen to ER. SERM-bound ER dimers interact with chromatin at EREs of the DNA. In the breast, they are associated with co-repressors (CoR) which inhibit ER transcriptional activity, but in other organ tissues such as bone and endometrium, they are associated with co-activators (CoA), allowing for gene transcription. *Selective estrogen receptor downregulators (SERDs)*. SERDs are pure ER antagonists. The SERD–ER complex is unable to translocate to the nucleus or undergo an open chromatin conformation that would allow transcription of ER-regulated genes. The SERD-ER complex subsequently undergoes proteosomal degradation. *Proteolysis targeting chimerics (PROTACs)*: PROTACs are bifunctional molecules that consist of a ligand that binds to a target protein (ER) and another ligand that binds to the E3 ubiquitin ligase. The interaction results in ubiquitination and degradation of the target protein through the ubiquitin-proteasome complex. *Complete estrogen receptor antagonists (CERANs)*. CERANs block both transcriptional activation domains (AF1 and AF2) of ER by recruiting nuclear receptor corepressors (N-CoR) to inactivate AF1 and directly inhibit AF2. *Selective estrogen receptor covalent antagonists (SERCAs)*. SERCAs covalently bind to a cysteine residue (C530) on ER, resulting in ER inactivation and inhibition of gene transcription.

A new generation of novel anti-estrogen therapies was designed to circumvent some of these resistance mechanisms, especially acquired *ESR1* mutations, and address limitations of current endocrine therapy, such as the agonist activity of tamoxifen and intramuscular administration of fulvestrant. These agents include variations of drug classes that already exist, including SERMs other than tamoxifen and novel orally administered SERDs. SERDs were initially identified as selective estrogen receptor down-regulators, but after studies confirmed that reduction in ER levels through proteasome-dependent degradation was responsible for their efficacy, they were termed degraders^22^. Novel anti-estrogen drug classes include complete estrogen receptor antagonists (CERANs), selective estrogen receptor covalent antagonists (SERCAs), and proteolysis-targeting chimerics (PROTACs) targeting ER. Each class of medication has a distinct mechanism of action, as illustrated in Fig. 1.

In this perspective, we will review novel anti-estrogenic agents being evaluated in breast cancer, including preliminary or final efficacy and safety results from some trials and ongoing and/or planned randomized phase II–III trials that will define whether they will have a potential role in the management of early and advanced stage breast cancer. The results of phase I trials evaluating various agents are summarized in Table 1, including the recommended phase II doses (RP2D) when used as monotherapy or in combination with CDK4/6 inhibitors. The efficacy of various novel agents in phase I or phase I–II trials are summarized in Table 2, which also includes the characteristics of the patient populations. Notably, several of these trial results have only been presented at national meetings in abstract form and published data in peer-reviewed journals is pending.

**Table 1:** Adverse events and recommended phase II doses of select anti-estrogen therapies.

Drug class/drug	DLTs in phase I	Recommended phase II monotherapy dose	Recommended phase II dose with CDK 4/6 inhibitors (i)	Common adverse events occurring in >10% (except where noted): monotherapy	Common adverse events occurring in >10%: combined with CDK 4/6i	References
*SERMs*						
Lasofoxifene	None	5 mg oral daily	5 mg oral daily	Fatigue, nausea, arthralgias, hot flashes	Diarrhea, nausea, leukopenia	Goetz et al.^32^ Damodaran et al.^33^
*SERM/SERD*						
Elacestrant (RAD1901)	None	400 mg oral daily	–	Nausea, fatigue, vomiting, decreased appetite, arthralgia	–	Bardia et al.^41^
*SERDs*						
Giredestrant (GDC-9545)	None	30 mg oral daily	100 mg oral daily	Fatigue, arthralgia, back pain, nausea, diarrhea, cough, constipation, pain in extremity	Neutropenia, fatigue, bradycardia, diarrhea, constipation, dizziness, nausea, anemia, asthenia, thrombocytopenia, pruritus, and visual impairment	Lim et al.^47^ Jhaveri et al.^46^
Camizestrant (AZD9833)	Monotherapy: QTc prolongation, vomiting, a combination of visual disturbance, headache, and gait disturbance Combination: visual disturbance	75 mg oral daily	75 mg oral daily	Visual disturbances, bradycardia, nausea, fatigue, dizziness, vomiting, and asthenia	Visual disturbances, bradycardia, asthenia, anemia, QTc prolongation, nausea, neutropenia, decreased white blood cell count, and vomiting	Hamilton et al.^60^ Baird et al.^61^
Imlunestrant (LY-348356)	None	400 mg oral daily	–	Nausea, diarrhea, fatigue, arthralgia, urinary tract infection, constipation, headache	–	Jhaveri et al.^71^
Rintodestrant (G1T48)	None	800 mg oral daily	800 mg oral daily	Hot flashes, fatigue, nausea, diarrhea, vomiting	Neutropenia, leukopenia, anemia, asymptomatic bacteriuria, thrombocytopenia	Aftimos et al.^75^ Maglakelidze et al.^76^
ZN-c5	None	Final dose pending (25 mg and 50 mg oral daily under discussion)	–	Hot flashes, nausea, fatigue	–	Kalinsky et al.^78^
D-0502	None	400 mg oral daily	Final dose pending (200 mg and 400 mg oral daily under discussion)	Nausea, diarrhea, vomiting, ALT increase, AST increase, fatigue, dizziness, anemia, hyperglycemia	Nausea, diarrhea, vomiting, abdominal pain, neutropenia, leukopenia, urinary tract infection, fatigue, dizziness, anemia, decreased appetite, vertigo	Osborne et al.^81^
*PROTAC*						
ARV-471	None	Final dose pending (200 mg and 500 mg oral daily under discussion)	–	Nausea, fatigue, vomiting, AST increase	–	Hamilton et al.^87^
*CERAN*						
OP-1250	None	60 mg to 120 mg oral daily	–	Occurring in > 15%: Nausea, fatigue, constipation, headache, vomiting, decreased appetite, neutropenia, maculopapular rash	–	Patel et al^92^
*SERCA*						
H3B-6545	None	450 mg oral daily	–	Nausea, fatigue, diarrhea, GFR decrease, anemia, lymphopenia, ALT increase, AST increase, bilirubin increase, creatinine increase, asymptomatic sinus bradycardia	–	Hamilton et al.^95^

**Table 2:** Efficacy of select single agent anti-estrogen therapies in phase I and phase I–II non-randomized studies.

Drug class/drug	Phase/clinical trial	*N*	Median lines for MBC	Prior CDK 4/6i	Prior fulvestrant	ESR1 mutation	ORR	CBR	PFS (months)	Reference
*SERM/SERD*										
Elacestrant (RAD1901)	Phase I RAD1901-005	50	3 (1–7)	52%	52%	50%	19.4%	42.6%	4.5	Bardia et al.^41^
*SERD*										
Giredestrant (GDC-9545)	Phase Ia/Ib	41	1 (0–2)	66%	20%	51%	20%	54%	7.2	Turner et al.^49^
Camizestrant (AZD9833)	Phase I SERENA-1	98	3 (0–7)	62%	53%	43%	10%	35.3%	5.4	Baird et al.^61^
Imlunestrant (LY-348356)	Phase I EMBER-1	114	2 (0–8)	92%	51%	49%	8%	42%	4.3	Jhaveri et al.^70^
Rintodestrant (G1T48)	Phase I	67	2 (0–9)	69%	64%	43%	5%	30%	2.6-3.6	Aftimos et al.^75^
ZN-c5	Phase I/II 565TiP	56	2 (0–9)	70%	46%	41%	5%	38%	3.8	Kalinsky et al.^78^
D-0502	Phase I	22	–	–	–	–	5%	36%	–	Osborne et al.^81^
*PROTAC*										
ARV-471	Phase I	60	4 (1–10)	100%	80%	–	13%	40%	–	Hamilton et al.^87^
*CERAN*										
OP-1250	Phase I/II	40	3 (1–6)	92.5%	67.5%	50%	9%	21%	–	Patel et al.^92^
*SERCA*										
H3B-6545	Phase II	94	3 (1–8)	87%	68%	62%	16.4%	39.7%	3.8	Hamilton et al.^97^

### Results of completed randomized phase II–III trials

Randomized trials with results reported are summarized in Table 3, including those for metastatic and localized breast cancer. In all of these trials, only patients with ER-positive and HER2-negative diseases were included. For some of these agents, other randomized trials (Table 4) and/or non-randomized trials (Table 5) are ongoing.

**Table 3:** Completed randomized phase II/III studies of select anti-estrogen therapies in metastatic and early breast cancer with results reported.

Drug	Clinical trial	*N*	Patient characteristics	Study design	Primary endpoints	Results	Reference
*Metastatic breast cancer*							
Lasofoxifene	Phase II ELAINE I	103	MBC ≥ second line including AI/CDK 4/6i with *ESR1* mutation	Lasofoxifene vs. fulvestrant	PFS	PFS in the overall population (*n* = 103) • No difference in two arms, HR 0.699 [CI: 0.445–1.125], *p* = 0.138 • Median PFS: 6.04 (lasofoxifene) vs. 4.04 (fulvestrant) months	Goetz et al.^32^
Elacestrant (RAD1901)	Phase III EMERALD	477	MBC with 1–2 prior lines of therapy, including CDK 4/6i	Elacestrant vs. standard of care (SOC) endocrine monotherapy (fulvestrant/AI)	PFS assessed by blinded independent central review (BICR) in • Overall population • Subset with *ESR1* mutation	PFS in overall population (*n* = 477) • Improved on elacestrant arm, HR 0.70 [CI: 0.55–0.88], *p* = 0.002 • 6-month PFS rates: 34.3% (elacestrant) vs. 20.4% (SOC) • 12-month PFS rates: 22.3% vs. 9.4% • Median PFS: 2.8 vs. 1.9 months, *p* = 0.0018 PFS in ESR1 mutant population (*n* = 228) • Improved on elacestrant arm, HR 0.55, [CI: 0.39–0.77], *p* = 0.0005 • 6-month PFS rates: 40.8% vs. 19.1% • 12-month PFS rates: 26.8% vs. 8.2% Interim overall survival analysis • Overall population (149 events): HR 0.75, [CI: 0.54–1.04], *p* = 0.08 • ESR1 mutant (68 events): HR 0.59, [CI: 0.36–0.96], *p* = 0.03, nonsignificant	Bidard et al.^42^
Giredestrant (GDC-9545)	Phase II acelERA	303	MBC with 1–2 prior lines of therapy, including at least 1 ET	Giredestrant vs. physician’s choice ET	PFS assessed by the investigator in the overall population	PFS in overall population (*n* = 303) • No difference in two arms, HR 0.81 [CI: 0.60–1.10], *p* = 0.18 • Median PFS: 5.6 (giredestrant) vs. 5.4 (physician’s choice ET) months PFS in ESR1 mutant population (*n* = 118) • No difference in two arms, HR 0.60 [CI: 0.35–1.03], *p* = 0.06 • Median PFS: 5.3 vs. 3.5 months	Martin Jiminez et al.^50^
*Early breast cancer*							
Giredestrant (GDC-9545)	Phase II coopERA	221	Untreated EBC and baseline Ki67 ≥ 5%	Window-of-opportunity phase with 14 days of giredestrant vs. anastrozole followed by 16 weeks of continued ET plus palbociclib	Ki67 change from baseline to week 2	Ki67 reduction from baseline to week 2 • Higher with giredestrant (mean reduction of 80% vs. 67%) Other outcomes • Ki67 reduction at surgery higher in giredestrant arm (81% vs. 74%) • ORR similar in both arms: 50% giredestrant vs. 49% anastrozole	Fasching et al.^55^

**Table 4:** Ongoing randomized phase II/phase III trials of novel anti-estrogen therapies.

Drug class	Drug	Clinical trial	Patient population	*N*	Design	Primary endpoint	Estimated completion date
EBC							
*SERD*	Giredestrant	Phase III lidERA NCT04961996	Medium- and high-risk EBC	4100	Monotherapy vs. physician’s choice of ET	IDFS	December 2025
*SERD*	Imlunestrant	Phase III EMBER-4	EBC with adjuvant ET for 2–5 years and increased risk of recurrence	TBD	Monotherapy vs. standard ET	To be decided	To be decided
MBC: 1st Line							
*SERD*	Giredestrant	Phase III persevERA NCT04546009	MBC 1st line	978	Combined with palbociclib vs. palbociclib + letrozole	PFS	April 2024
*SERD*	Camizestrant	Phase III SERENA-4 NCT04711252	MBC 1st line	1402	Combined with palbociclib vs. palbociclib + AI	PFS	November 2025
MBC: ≥2nd Line							
*SERD*	Giredestrant	Phase III evERA NCT05306340	MBC 2nd/3rd line	320	Combined with everolimus vs. everolimus + exemestane	PFS	July 2024
*SERD*	Giredestrant	Phase II acelERA NCT04576455	MBC 2nd/3rd line	303	Monotherapy vs. physician’s choice of ET	PFS	February 2022
*SERD*	Giredestrant	Phase Ib/II MORPHEUS NCT04802759	MBC 2nd/3rd line	415	Combined with abemaciclib, palbociclib, ribociclib, ipatasertib, inavolisib, everolimus, or samuraciclib	ORR	October 2026
*SERD*	Camizestrant	Phase I SERENA-1 NCT03616587	MBC ≥ 2nd line	305	Combined with abemaciclib, everolimus or capivasertib	DLT	December 2022
*SERD*	Camizestrant	Phase II SERENA-2 NCT04214288	MBC ≥ 2nd line	240	Monotherapy vs. fulvestrant	PFS	September 2022
*SERD*	Camizestrant	Phase III SERENA-6 NCT04964934	MBC on 1st line AI + CDK 4/6 inhibitor with detectable *ESR1* mutation but no disease progression	302	Combined with CDK 4/6 inhibitor vs. continue AI + CDK 4/6 inhibitor	PFS	September 2023
*SERD*	Imlunestrant	Phase III EMBER-3 NCT04975308	MBC ≥ 2nd line	800	Monotherapy vs. combined with abemaciclib vs. investigator’s choice ET	PFS	June 2023

**Table 5:** Ongoing non-randomized trials of novel anti-estrogen therapies.

Drug class	Drug	Clinical trial	Patient population	*N*	Design	Primary endpoint	Estimated completion date
EBC							
*SERM/SERD*	Elacestrant	Phase 0 ELIPSE NCT04797728	EBC without prior therapy	24	Single-agent preoperatively	Complete cell cycle arrest (Ki67 ≤ 2.7%)	March 2022
*SERD*	Camizestrant	Phase II SERENA-3 NCT04588298	EBC without prior therapy	92	Single-agent preoperatively	Change in ER expression	March 2023
*SERD*	Imlunestrant	Phase I EMBER-2 NCT04647487	EBC without prior therapy	90	Single-agent preoperatively	Change in ER expression	October 2022
MBC: ≥2nd line							
*SERM*	Lasofoxifene	Phase II ELAINEII NCT04432454	MBC ≥ 2nd line (prior AI/CDK 4/6i required) with *ESR1* mutation	29	Combined with abemaciclib	Safety (number and severity of AEs)	November 2022
*SERM/SERD*	Elacestrant	Phase Ib/II NCT04791384	MBC ≤ 2 lines of chemotherapy, any ET, and with brain metastases	44	Combined with abemaciclib	Overall intracranial response rate	January 2023
*SERD*	Giredestrant	Phase Ib/II MORPHEUS NCT04802759	MBC 2nd/3rd line	415	Combined with abemaciclib, palbociclib, ribociclib, ipatasertib, inavolisib, everolimus, or samuraciclib	ORR	October 2026
*SERD*	Camizestrant	Phase I SERENA-1 NCT03616587	MBC ≥ 2nd line	305	Combined with abemaciclib, everolimus or capivasertib	DLT	December 2022
*SERD*	Imlunestrant	Phase I/II EMBER-1 NCT04188548	MBC, HER2-positive or negative	500	Monotherapy and combined with alpelisib, abemaciclib, everolimus, trastuzumab or abemaciclib and trastuzumab	DLT	December 2023
*SERD*	Rintodestrant	Phase I NCT03455270	MBC ≥ 2nd line	107	Combined with palbociclib	RP2D	May 2022
*SERD*	ZN-c5	Phase Ib 564TiP NCT04514159	MBC ≥ 2nd line, no prior CDK 4/6 inhibitor	14	Combined with abemaciclib	MTD	April 2023
*SERD*	ZN-c5	Phase I/II 565TiP NCT03560531	MBC ≥ 2nd line	181	Combined with palbociclib	MTD	April 2022
*SERD*	Borestrant	Phase I/II ENZENO NCT04669587	MBC any line	106	Monotherapy and combined with palbociclib	RP2D	January 2023
*SERD*	D-0502	Phase I NCT03471663	MBC ≥ 2nd line	200	Monotherapy and combined with palbociclib	DLT	July 2023
*PROTAC*	ARV-471	Phase I/II NCT04072952	MBC ≥ 2nd line	215	Monotherapy and combined with palbociclib	DLT	September 2022
*CERAN*	OP-1250	Phase I/II NCT04505826	MBC ≥ 2nd line	94	Monotherapy	MTD	October 2023
*SERCA*	H3B-6545	Phase I NCT04288089	MBC ≥ 3rd line	36	Combined with palbociclib	MTD	March 2024

### Selective estrogen receptor modulators

SERMs display ER antagonist or agonist activity, depending on the cell type, through the recruitment of different co-activators and co-repressors. SERMs inhibit activating function domain 2 (AF2) of ER but allow for agonist signaling through activating function domain 1 (AF1) through other signaling pathways such as mTOR, PI3K, and MAPK. Tamoxifen was the first approved SERM and is now widely used in the adjuvant and metastatic settings for breast cancer based on randomized Phase III trials^23,24^. Evidence of superior efficacy of AIs and the side effect profile of tamoxifen has decreased the enthusiasm for this class though other SERMs are currently in development^6,7^. Raloxifene, another SERM, was as effective as tamoxifen in breast cancer prevention in high-risk women without increasing the risk of endometrial cancer in the National Surgical Adjuvant Breast and Bowel Project (NSABP) Study of Tamoxifen and Raloxifene trial^25^. Toremifene is a SERM with a structure and efficacy nearly identical to that of tamoxifen. The drug was initially developed to allow for an improved side effect profile though studies have not demonstrated any safety advantage for toremifene^26,27^. The SERM arzoxifene showed initial promising efficacy and favorable safety with antiestrogenic effects on both breast and endometrium, but phase III data found it to be inferior to tamoxifen, ending further clinical development^28,29^.

#### Lasofoxifene

Lasofoxifene is a next-generation non-steroidal SERM that differs from other SERMs based on its binding affinity, which is similar to 17β-estradiol, and strong preclinical data in ER-mutated breast cancer models which are resistant to AIs^30^. Lasofoxifene first demonstrated a reduction in the risk of both fractures and breast cancer in patients in the post-menopausal evaluation and risk-reduction with the Lasofoxifene (PEARL) trial^31^. Subsequently, in *ESR1* mutant models, lasofoxifene was shown to inhibit tumor growth at primary and metastatic sites compared to fulvestrant^30^. ELAINE I (NCT03781063) assessed the efficacy of lasofoxifene vs. fulvestrant in 103 patients, both pre- and postmenopausal, with MBC who have *ESR1* mutations and progressed on prior AI and CDK 4/6 inhibitors (Table 3). Results showed numerically improved PFS with lasofoxifene compared with fulvestrant (6.04 vs 4.04 months; hazard ratio 0.699, *p* = 0.138), though this did not reach statistical significance^32^. ELAINE II (NCT04432454) is an ongoing, non-randomized phase II study evaluating lasofoxifene in combination with abemaciclib (Table 5)^33^.

### Selective estrogen receptor degraders

Although SERMs inhibit ER through changes in ER structure and cofactor recruitment and AIs effectively reduce estrogen levels, the presence of ER itself can allow tumor to escape from ET and activate the ER signaling pathway. Progression in ER-positive breast cancer ultimately results from ligand-independent activation either through direct mutation of ER or phosphorylation of ER or its coregulators through signaling pathways such as PI3K-AKT-mTOR. SERDs address some of these resistance mechanisms, unlike SERMs and AIs, as they function not only as competitive ER antagonists but also induce proteasome-dependent degradation of ER^34^. Fulvestrant is the prototype of the SERD class and is currently the only approved SERD for the treatment of ER-positive MBC. The promising efficacy of fulvestrant has fueled interest in the SERD approach and steered the advancement of numerous orally bioavailable SERDs.

#### Fulvestrant

Several randomized trials have established the efficacy of fulvestrant as a single agent and in combination with various biologic and targeted agents. A meta-analysis of 11 trials including 5808 patients found that fulvestrant 500 mg was superior to fulvestrant 250 mg, megestrol acetate, and anastrozole, with regard to PFS^35^. In the phase III FALCON trial, women with ER-positive MBC without prior ET were randomized to either fulvestrant or anastrozole. The primary endpoint of PFS was increased in the fulvestrant arm (16.6 months) compared to the anastrozole arm (13.8 months)^36^. In phase III randomized trials in patients with metastatic ER-positive MBC, fulvestrant has demonstrated increases in PFS when combined with targeted agents such as CDK 4/6 inhibitors, alpelisib, and everolimus^11–13,16,37^.

Particularly, limitations of fulvestrant include its intramuscular administration. This has prompted the search for alternative orally bioavailable SERDs, which are currently under evaluation in clinical trials for use in metastatic, adjuvant, and neoadjuvant settings. Herein, we describe the development, toxicity profile, and corresponding trial for each novel SERD.

#### Elacestrant (RAD1901)

Elacestrant is an orally bioavailable SERM/SERD hybrid that is furthest along in development at this time. The drug functions as a partial agonist at lower doses and as an antagonist at higher doses. As receptor occupancy increases, degradation occurs, resulting in the inhibition of *ESR1* signaling^38^. Elacestrant first demonstrated anti-tumor activity in breast cancer patient-derived xenograft (PDX) models, including those harboring *ESR1* mutations^39,40^. These preclinical studies formed the basis for a Phase I study of elacestrant monotherapy in patients with heavily pretreated ER-positive MBC. The drug demonstrated antitumor activity and tolerability, and the trial established the RP2D at 400 mg once daily (Table 1), with nausea, fatigue, vomiting, anorexia, and arthralgias being the most common adverse effects^41^. Objective response was observed in 19.4% of patients, of whom at least half had prior fulvestrant (54%), CDK 4/6 inhibitors (52%), and *ESR1* mutations (50%, Table 2).

The ensuing phase III EMERALD trial (NCT03778931) included postmenopausal patients with ER-positive, HER2-negative MBC with prior CDK 4/6 inhibitor therapy, 1–2 lines of ET, and ≤1 chemotherapy (Table 3). A total of 477 patients were randomly assigned to elacestrant 400 mg orally once daily or standard-of-care (SOC) endocrine monotherapy, which included either fulvestrant or an AI. Approximately 48% of patients had detectable *ESR1* mutations. The results revealed prolonged PFS in the intention-to-treat (ITT) population receiving elacestrant with 12-month PFS rates of 22.3% vs. 9.4% in patients on elacestrant versus SOC with a hazard ratio (HR) of 0.70 (0.55–0.88). A greater magnitude of benefit was observed in the subgroup of patients with tumors harboring *ESR1* mutations with HR 0.55 (0.39-0.77). Notably, the absolute PFS benefit in the study was small (2.8 vs. 1.9 months in the overall population), and this was attributed to rapid progression in the majority of patients in both treatment arms, after which the PFS curves diverged. An interim OS analysis (149 deaths) performed at the time of the prespecified final PFS analysis revealed a trend favoring elacestrant in the overall population (HR 0.75, 95% CI: 0.54–1.04, *p* = 0.08) and *ESR1* mutant population (HR 0.59, 95% CI: 0.36–0.96, *p* = 0.03), but not the *ESR1* non-mutant population (HR 0.92, 95% CI: 0.59–1.42, *p* = 0.69). Final OS analysis is expected when approximately 50% of the study population has died (239 deaths). Regarding safety, 27% of patients on elacestrant experienced a Grade 3/4 AE, such as nausea, back pain, and increased ALT, compared with 20.5% on the SOC arm. There were no treatment-related deaths^42^.

EMERALD was the first phase III trial evaluating an oral SERD against SOC endocrine therapy in patients with MBC and previous treatment with CDK 4/6 inhibitor. The higher magnitude of response in the subset with *ESR1* mutations highlights the potential use of *ESR1* as a predictive biomarker for this drug and other novel anti-estrogen agents. In January 2023, the U.S. Food and Drug Administration (FDA) approved elacestrant for patients with ER-positive, HER2-negative, and ESR1 mutated MBC following at least 1 line of ET. Elacestrant is also currently being evaluated in combination with abemaciclib in patients with brain metastases (NCT04791384) and in the presurgical setting by assessing change in Ki67 (NCT04797728, Table 5)^43,44^.

#### Giredestrant (GDC-9545)

Giredestrant is another orally bioavailable SERD that first demonstrated antitumor activity as a single agent and in combination with a CDK 4/6 inhibitor in PDX models^45^. A subsequent phase I a/b study (NCT03332797) evaluated giredestrant monotherapy (30 mg oral daily) and combination therapy (100 mg daily) with palbociclib in postmenopausal patients with ER-positive MBC who had disease recurrence while on adjuvant ET for ≥24 months or progression after prior ET for ≥6 months and ≤2 lines of therapy (Table 1). Drug tolerance and clinical activity were observed as a single agent and in combination with palbociclib. Most common AEs with giredestrant monotherapy included fatigue, arthralgias, and nausea, and only 5% of patients had Grade 3 AEs. Of note, 7% had bradycardia, but these were Grade 1–2 events. Among patients on combination therapy, 57% had Grade ≥3 AEs, the most common of which was neutropenia. Thirteen percent of 85 patients had Grade 1 asymptomatic bradycardia^46–48^. In terms of efficacy, ORR was 20% in 41 patients on single-agent giredestrant (Table 2) and 38% in 44 patients on giredestrant and palbociclib combination. CBRs were 54% and 81% in 41 patients on monotherapy and 48 on combination, respectively. Paired pre- and on-treatment biopsies from 21 patients illustrated consistent downregulation of ER, PR, Ki67, and ER pathway activity as measured by gene expression analysis on Cycle 2 Day 8. Thirty-four of 36 patients (94%) with detectable baseline ctDNA *ESR1* level had a decrease after 4 weeks of therapy^47,49^.

The encouraging activity of giredestrant in Phase I studies has led to several phase II/III studies in the metastatic and early-stage settings. acelERA (NCT04576455) was a randomized phase II study evaluating the efficacy and safety of giredestrant versus physician’s choice of ET in postmenopausal and premenopausal women on ovarian function suppression (OFS) with ER-positive, HER2-negative, advanced/MBC who have received 1–2 prior lines of systemic therapy, at least one of which was ET (Table 3). Interim analysis in 303 patients with a median follow-up of 7.89 months showed no significant improvements in PFS in the overall population, although there was a non-significant trend for benefit in the *ESR1* mutant subgroup (median PFS 5.3 vs. 3.5 months; HR 0.60 [CI: 0.35–1.03], *p* = 0.06)^50^.

persevERA (NCT04546009) is an ongoing Phase III double-blind, placebo-controlled, randomized trial evaluating the efficacy and safety of giredestrant and palbociclib versus letrozole and palbociclib in patients with ER-positive, HER2-negative MBC in the first line setting (Table 4)^51^. The Phase III randomized evERA trial (NCT05306340), which evaluates the efficacy of giredestrant and everolimus compared with exemestane and everolimus, is also ongoing (Table 4)^52^. A randomized umbrella trial (NCT04802759) is assessing the efficacy of giredestrant in combination with CDK 4/6 inhibitors, ipatasertib, inavolisib, everolimus, and samuraciclib among others (Table 4)^53^.

In the early-stage setting, the phase II coopERA BC trial (NCT04436744) randomized postmenopausal women with untreated ER-positive early breast cancer (EBC) and baseline Ki67 ≥ 5% to receive preoperative giredestrant versus anastrozole for a 14-day window-of-opportunity phase followed by 16 weeks of continued ET in addition to palbociclib (Table 3). The primary endpoint of Ki67 change from baseline to Week 2 was higher with giredestrant (mean reduction in Ki67 of 80%) compared with anastrozole (mean reduction of 67%)^54^. Final analyses in 221 patients showed that Ki67 suppression at surgery remained higher in the giredestrant arm (81% versus 74%). ORR was similar in both arms (50% giredestrant versus 49% anastrozole)^55^. The randomized phase III lidERA trial (NCT04961996) will evaluate adjuvant giredestrant vs. physician’s choice of ET for at least 5 years in patients with medium- and high-risk ER-positive EBC. The primary endpoint is invasive disease-free survival (IDFS) with a target enrollment of 4100 patients (Table 4)^56^.

#### Amcenestrant (SAR-439859)

Amcenestrant is another oral SERD that was investigated in several clinical trials, but after a recent phase III study comparing amcenestrant plus palbociclib with letrozole plus palbociclib demonstrated an advantage for the letrozole plus palbociclib arm, Sanofi decided to end clinical development of amcenestrant, and other ongoing studies were discontinued^57,58^.

#### Camizestrant (AZD9833)

Camizestrant is an oral SERD that showed tumor growth suppression in PDX models, including those with *ESR1* mutations^59^. The phase I SERENA-1 trial (NCT03616587) investigated camizestrant as monotherapy and in combination with palbociclib in postmenopausal and premenopausal women on OFS with advanced HR-positive BC after ≥1 ET and ≤2 chemotherapies (Table 1). In the monotherapy dose escalation phase, at dose levels from 25 to 450 mg daily, 3 patients experienced dose-limiting toxicities (DLTs), including Grade 3 QTc prolongation, Grade 3 vomiting, and a combination of Grade 2 visual disturbance, headache, and gait disturbance, all of which resolved with dose reduction. No Grade 4 or 5 AEs were reported. Most common TRAEs included visual disturbances, bradycardia, nausea, and fatigue, among others. The 75 mg dose was subsequently established as the RP2D^60^. In a heavily pretreated population, camizestrant demonstrated clinical activity as monotherapy with ORR of 10% and CBR of 35.3% across all dose levels and CBR of 53.3% and PFS of 11.1 months in patients on the 75 mg dose (Table 2). When studied in combination with palbociclib, the toxicity profile was overall similar to camizestrant monotherapy with two DLTs (Grade 3 QTc prolongation and Grade 2 visual disturbances), both of which resolved with dose interruption and reduction^61^. Updated analyses of the dose expansion cohort of camizestrant 75 mg daily and palbociclib in 48 patients revealed an ORR of 6.3% and CBR of 50%^62^. The trial is also evaluating the drug in combination with abemaciclib, everolimus, and capivasertib^63^.

Several additional studies with camizestrant are ongoing or planned in MBC (Table 4). SERENA-2 (NCT04214288) is a randomized phase II trial comparing efficacy and safety of three dose levels of camizestrant vs. fulvestrant in a population that has progressed after at least 1 ET^64^. There are two ongoing phase III randomized trials in the first-line metastatic setting. SERENA-4 (NCT04711252) is comparing camizestrant in combination with palbociclib versus AI and palbociclib^65^. SERENA-6 (NCT04964934) is enrolling patients who have received first-line AI and CDK 4/6 inhibitor (palbociclib or abemaciclib) for at least 6 months without progression and are monitored regularly for the presence of *ESR1* mutations via ctDNA analysis; those with detectable *ESR1* mutations without disease progression are randomized to either continue AI and CDK 4/6 inhibitor or switch ET to camizestrant and continue the same CDK 4/6 inhibitor^66^.

In the window-of-opportunity SERENA-3 trial (NCT04588298), postmenopausal women with newly diagnosed ER-positive EBC will be randomized to receive 75 mg or 150 mg oral camizestrant for 5–7 days prior to surgery (Table 5). The study will evaluate the drug’s effect on ER expression in pre- and on-treatment tumor samples^67^.

#### Imlunestrant (LY-3484356)

Imlunestrant is an oral SERD that demonstrated promising efficacy in the preclinical setting with potent inhibition of *ESR1* wildtype and mutant xenograft tumors. Synergistic effects were observed when combined with abemaciclib, everolimus, and alpelisib^68^. The Phase I/II EMBER-1 trial (NCT04188548) is evaluating the drug as a single agent and in combination with alpelisib, abemaciclib, everolimus, trastuzumab or abemaciclib and trastuzumab in postmenopausal and premenopausal women on OFS who have advanced ER-positive BC and endometrial endometrioid cancer (Table 5)^69^. Data from the dose escalation and dose expansion cohort of 114 patients on imlunestrant monotherapy demonstrated a favorable safety profile and encouraged anti-tumor activity (Table 1). No DLTs were observed. Most treatment-emergent adverse events (TEAEs) were Grade 1 and included nausea, diarrhea, fatigue, and arthralgia. Grade ≥3 TRAEs occurred in 7 patients. In terms of efficacy, the ORR was 8%, and CBR was 42% (Table 2). Complete clearance or decline in *ESR1* ctDNA levels was observed for 73% of 44 patients with baseline *ESR1* mutations. While the median PFS for the overall population was 4.3 months, for patients on second-line imlunestrant, it was 6.5 months^70^. EMBER-3 (NCT04975308) is a phase III randomized study of imlunestrant monotherapy, investigator’s choice ET, or imlunestrant plus abemaciclib in patients with ER-positive MBC previously treated with ET (Table 4)^71^.

In the early-stage setting, EMBER-2 (NCT04647487) is investigating the biological effects of pre-operative imlunestrant by evaluating changes in ER expression (Table 5)^72^. A phase III trial, EMBER-4, is being planned to evaluate the benefit of adjuvant imlunestrant vs. standard adjuvant ET in patients with ER-positive EBC, with prior adjuvant ET for 2–5 years and an increased risk of recurrence (Table 4)^70^.

### Other agents with no data from randomized trials

Other agents that have undergone phase I or phase I–II evaluation with safety data (Table 1) and preliminary efficacy data (Table 2) are summarized here, some of which include agents that are further being evaluated in ongoing randomized (Table 4) and non-randomized (Table 5) clinical trials.

### Selective estrogen receptor degraders (SERDs)

#### Rintodestrant (G1T48)

Rintodestrant is a novel orally bioavailable SERD that has demonstrated potent tumor inhibition in animal models with tamoxifen resistance and *ESR1* mutations^73^. A phase I study (NCT03455270) evaluated rintodestrant in pre- and postmenopausal women with HR-positive MBC after progression on ET. In the dose escalation phase, the drug demonstrated target engagement on ^18^F-fluoroestradiol positron emission tomography (FES-PET), a tolerable side effect profile, and antitumor activity in heavily pretreated patients (Table 1)^74^. In the dose expansion portion, among 67 patients with a median of 2 prior lines of therapy, ORR was 5% and CBR 30% (Table 2). The activity was observed regardless of *ESR1* or *PIK3CA* mutation status. Most common TRAEs included hot flashes, fatigue, nausea, diarrhea, and vomiting, most of which were Grade 1–2. Serious TRAEs included 1 patient with Grade 5 cerebral hemorrhage and another with Grade 2 upper abdominal pain. Two patients discontinued treatment due to TRAEs. No DLTs were observed^75^. Part 3 of this trial is assessing rintodestrant 800 mg daily with palbociclib in a population with prior ET but no prior CDK 4/6 inhibitor therapy. Preliminary data in 40 patients revealed an ORR of 5% and a CBR of 60%. The combination was well tolerated, with most common AEs related to the known safety profiles of palbociclib and rintodestrant^76^.

#### ZN-c5

ZN-c5 is a novel, small-molecule SERD with high oral bioavailability. In preclinical studies, the drug resulted in tumor growth inhibition which was enhanced when combined with CDK 4/6 inhibitors or PI3K inhibitors. ZN-c5 also showed increased efficacy in *ESR1* PDX models when compared to fulvestrant^77^. 565TiP is a phase I/II study (NCT03560531) evaluating ZN-c5 as monotherapy and in combination with palbociclib in postmenopausal and premenopausal women on OFS with advanced ER-positive BC with a prior response on ET for at least 6 months. Results from 45 evaluable subjects in the dose escalation and expansion cohorts with single agent ZN-c5 demonstrated no DLTs, with the most common TRAEs including hot flashes, nausea, and fatigue; grade 3 TEAEs included abdominal pain, hypertension, hyponatremia, pain in extremities, and GGT increase (Table 1). With regard to efficacy in this population, the ORR was 5% and CBR 38% (Table 2). Phase II testing of Zn-c5 monotherapy and phase I testing of combination with palbociclib are in progress (Table 5)^78^. Recruitment for 564TiP, a phase 1b trial (NCT04514159) of ZN-c5 combined with abemaciclib in patients without prior CDK 4/6 inhibitors, is ongoing as well (Table 5)^79^.

#### D-0502

D-0502 is another orally bioavailable SERD with anti-tumor activity in PDX models, including those with *ESR1* mutations^80^. A Phase I trial (NCT03471663) is investigating D-0502 as monotherapy and in combination with palbociclib to identify the RP2D in postmenopausal and premenopausal women on OFS who have HR-positive MBC. In the dose escalation portion, no DLTs and a favorable safety profile were observed. The most common AEs included nausea, vomiting, diarrhea, fatigue, alanine aminotransferase elevation, and neutropenia (Table 1). Preliminary efficacy results showed ORR of 5% and CBR of 36% in 22 patients on monotherapy (Table 2). Among 13 patients on D-0502 plus palbociclib, ORR and CBR were 15% and 77%, respectively. Future efficacy results from the dose expansion cohort will be informative^81^.

#### Borestrant (ZB-716)

Borestrant is a boronic acid-modified fulvestrant with oral bioavailability. It has demonstrated the downregulation of ER in endocrine-resistant breast cancer cells and superior tumor inhibition when compared to fulvestrant in PDX models^82^. ENZENO (NCT04669587) is an ongoing first-in-human study evaluating the safety and tolerability of ZB-716 as a single agent and in combination with palbociclib in patients with ER-positive MBC (Table 5)^83^.

### Proteolysis targeting chimerics (PROTACs)

PROTACs are bifunctional hybrids that simultaneously bind to a specific target protein, such as ER, and an E3 ubiquitin ligase resulting in ubiquitination and degradation of the target protein ER through the ubiquitin-proteasome system^84^. As their mechanism of action is catalytic, they are able to promote protein degradation even at low exposure levels. PROTAC technology has been adapted to target ER with several PROTACs in development, the furthest along of which is ARV-471^85^. In PDX models with and without *ESR1* mutations, oral daily administration of ARV-471 resulted in tumor regression^86^. A Phase I first-in-human study of ARV-471 enrolled postmenopausal patients with ER-positive MBC that had progressed on ≥2 lines of ET and ≥1 CDK 4/6 inhibitor; the drug was tolerated well with no DLTs, and the most common AEs were nausea, fatigue, and vomiting (Table 1). Patients were heavily pretreated with a median of 4 prior therapies; all had received prior CDK 4/6 inhibitor, and 80% had previous fulvestrant. Of 47 evaluable patients, the CBR was 40% (Table 2)^87^. The phase II dose escalation portion of this study is ongoing. A Phase I/II clinical trial (NCT04072952) of the combination of ARV-471 and palbociclib in this patient population is also ongoing (Table 5)^88^.

### Complete estrogen receptor antagonists (CERANs)

The estrogen receptor includes two distinct transcriptional activation domains, AF1, which is activated by signaling pathways such as mTOR, PI3K, and MAPK, among others, and AF2, which is activated by the estrogen ligand itself. Activation of AF1 and AF2 both lead to gene transcription and cell proliferation. CERANs block both AF1 and AF2 transcriptional activation domains of ER. CERANs directly inhibit AF2 and recruit nuclear receptor corepressors (N-CoR) to inactivate AF1. This differs from SERMs which inhibit AF2 but allow agonist signaling via AF1 through other signaling pathways^89^.

OP-1250 is an orally bioavailable CERAN that also acts as a SERD-inducing ER degradation. In preclinical studies, OP-1250 demonstrated blockade of both wild-type and mutant ER, inhibition of estrogen-stimulated proliferation in breast cells as well as receptor degradation. In xenograft models, OP-1250 resulted in shrinkage of breast tumors expressing both wild-type and mutant ER^90^. Nonclinical studies have also demonstrated activity in mutant *ESR1* tumors in the brain^91^. A phase I/II first-in-human study (NCT04505826) is evaluating the safety and tolerability of OP-1250 in postmenopausal and premenopausal women on OFS who have HR-positive MBC with progression on prior ET. No DLTs were observed, and most TEAEs were Grade 1–2, with the most common being nausea, fatigue, and constipation (Table 1). Phase 1b dose expansion and Phase 2 efficacy evaluation are ongoing. Preliminary data in 40 subjects with a median of 3 prior lines of therapy demonstrated anti-tumor activity (ORR 9%, CBR 21%) and drug tolerability. In the cohort within the anticipated RP2D range, ORR was 18% (2/11), and CBR was 38% (3/8) (Table 2)^92^.

### Selective estrogen receptor covalent antagonists (SERCAs)

SERCAs inactivate ER by engaging a unique cysteine residue that is not present in other hormone receptors^93^. HRB-6545 is a first-in-class SERCA that covalently binds to a cysteine residue at position 530 of both wild-type and mutant ER proteins. The novel drug was found to antagonize both wild-type and mutant ER in in vitro studies. In xenograft models, the small molecule showed superior antitumor activity compared to fulvestrant^94^. In Phase I/II study (NCT03250676) of single-agent H3B-6545, pre- and postmenopausal women with previously treated locally advanced or metastatic HR-positive BC tolerated the drug well, and no DLTs were observed (Table 1). Of note, 35% of patients experienced Grade 1 asymptomatic sinus bradycardia and 5% with Grade 2 symptomatic bradycardia without requiring intervention. Other common AEs included nausea, fatigue, diarrhea, glomerular filtrate rate (GFR) decrease, hemoglobin decrease, and lymphocyte decrease. Serious AEs were reported in 21% of patients and led to treatment discontinuation in 13% of patients. In the evaluable population of 94 patients who had a median of 3 prior lines of therapy, and most were previously treated with CDK 4/6 inhibitor, preliminary analyses showed an ORR of 16.4%, CBR of 39.7%, and median PFS of 3.8 months (Table 2). The response was seen in patients with visceral metastases, heavily pretreated disease, and *ESR1* mutations^95–97^. H3B-6545 is also being studied in combination with palbociclib in patients with HR-positive MBC with 2 or more prior therapies (NCT04288089, Table 5)^98^.

A new generation of anti-estrogen therapies, including SERMs other than tamoxifen and novel orally administered SERDs, and novel agents such as CERANs, SERCAs, and PROTACs targeting ER are being actively developed, driven primarily by the quest to develop agents that circumvent mechanisms of primary and secondary resistance to ET. Results thus far have been mixed, with statistically significant but clinically modest benefits observed with the oral SERD elacestrant when used in ET-resistant disease, especially when associated with *ESR1* mutations, and clear failures with the oral SERD amcenestrant when used as first-line or second-line ET. Preliminary results with other oral SERDs, such as giredestrant have also suggested some potential benefits in patients with tumors harboring *ESR1* mutations. At least 3 oral SERDs thus far, including giredestrant, imlunestrant, and camizestrant, are being evaluated in phase III trials in metastatic and/or early breast cancer. Other novel agents, including CERANs, SERCAs, and PROTACs, are in the early phases of clinical development, with some expected to be further evaluated in phase III trials. Although efficacy data from Phase III trials will guide their incorporation into clinical practice, the optimal sequencing and combinations of these drugs with other agents will pose additional opportunities for drug development. Key factors that will influence their impact on practice include drug tolerability, efficacy combined with or after targeted therapies such as CDK 4/6 inhibitors, mTOR inhibitors, and PI3K inhibitors, activity in patients with *ESR1* mutations, and differential ability to cross the blood-brain barrier. These considerations will impact whether these novel therapies will exceed existing ET options, including tamoxifen, aromatase inhibitors, and fulvestrant.

### Reporting summary

Further information on research design is available in the Nature Portfolio Reporting Summary linked to this article.

## Supplementary information

Reporting Summary Checklist
